# Epidemiology of Chronic Hepatitis B in Turkey

**DOI:** 10.5005/jp-journals-10018-1264

**Published:** 2018-05-01

**Authors:** Hasan Özkan

**Affiliations:** Department of Gastroenterology, School of Medicine, Ankara University, Ankara, Turkey

**Keywords:** Hepatitis B virus, Management, National support, Prevalence.

## Abstract

Hepatitis B virus (HBV) infection is a global public health problem and it is also a major health concern of Turkey. The estimated number of HBV carriers in Turkey is about 3.3 million, with an overall HBV prevalence of 4.57%. Thus, both prevention and therapy of HBV-infected patients are urgent medical need of Turkey. A total of 1,533 among 37,637 patients who were examined at the Department of Gastroenterology, Ankara University School of Medicine were found to be hepatitis B surface antigen (HBsAg) positive (4%). Viral hepatitis treatment is fully reimbursed in Turkey through the national insurance system, which covers all residents across Turkey.

**How to cite this article:** Özkan H. Epidemiology of Chronic Hepatitis B in Turkey. Euroasian J Hepato-Gastroenterol 2018;8(1):73-74.

## INTRODUCTION

Hepatitis B is one of the most common infectious diseases worldwide. Estimates indicate that almost 2 billion people have been infected with HBV, with more than 380 million people being chronic carriers (6% of the world population on average).

Among all chronic hepatitis B (CHB) cases, approximately 40% will develop cirrhosis, liver failure, or hepatocellular carcinoma. About 750,000 patients per year die from these complications.

According to the World Health Organization, Turkey has intermediate (2-8%) endemicity for hepatitis B. This information has been obtained mainly from studies in blood donors (Graph The overall prevalence of the hepatitis B surface antigen (HBsAg) has been previously reported to be in the range of 4.0 to 5.0%, which has decreased to 2.0% in recent years.

Since the implementation of universal vaccination of all children and risk groups in Turkey in 1998, a decline in prevalence has been observed. The prevalence across Turkey among children was observed before the vaccination policy. Before 1998, the overall country prevalence was found to be 5.90% among 0- to 15-year-olds. In the aftermath of the vaccination policy, this figure dropped to 2.84% for the same age group.^2^

**Graph 1:**
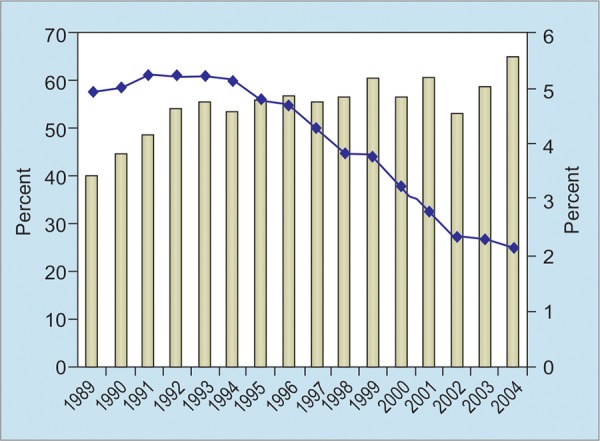
The prevalance of HBV in blood donors in Turkey^1^

The Turkish Society of Viral Hepatitis researched the prevalence of HBV among 73,175 people. The HBsAg positivity was found to be 2.3% with predominant D (HDV) genotype in different parts of Turkey in 2012.

**Fig. 1:**
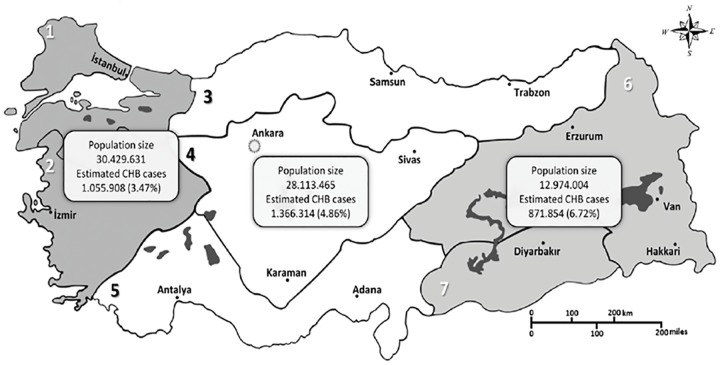
Distribution of HBV infection in different parts of Turkey^3^

**Graph 2:**
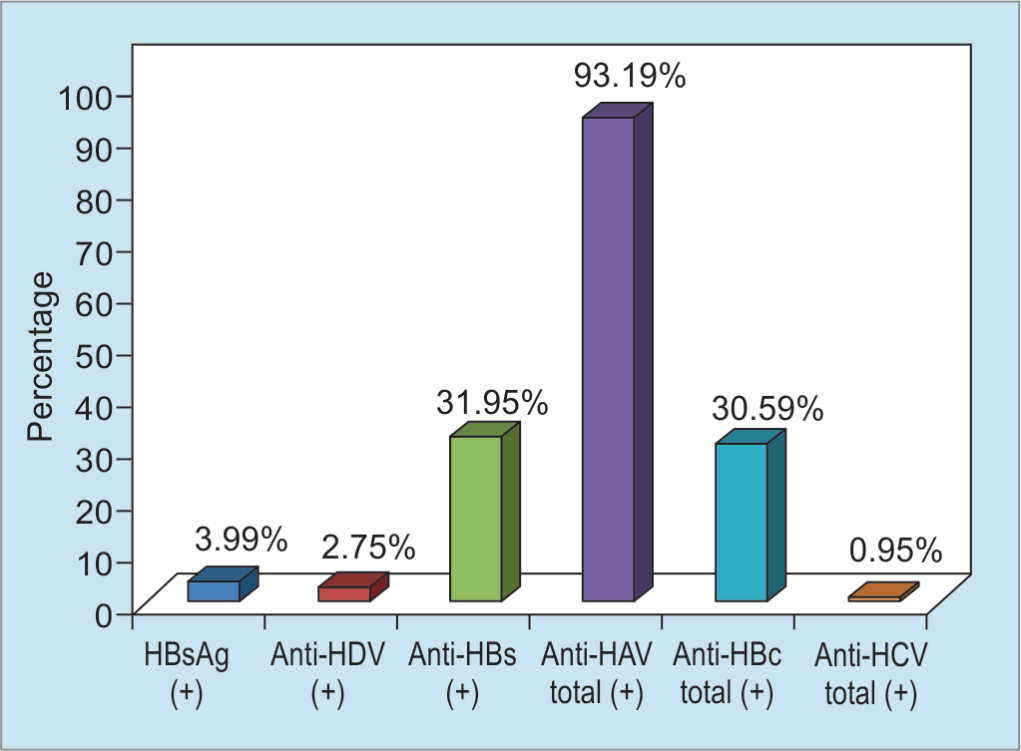
Health situation of HBV in Turkey^4^

**Graph 3:**
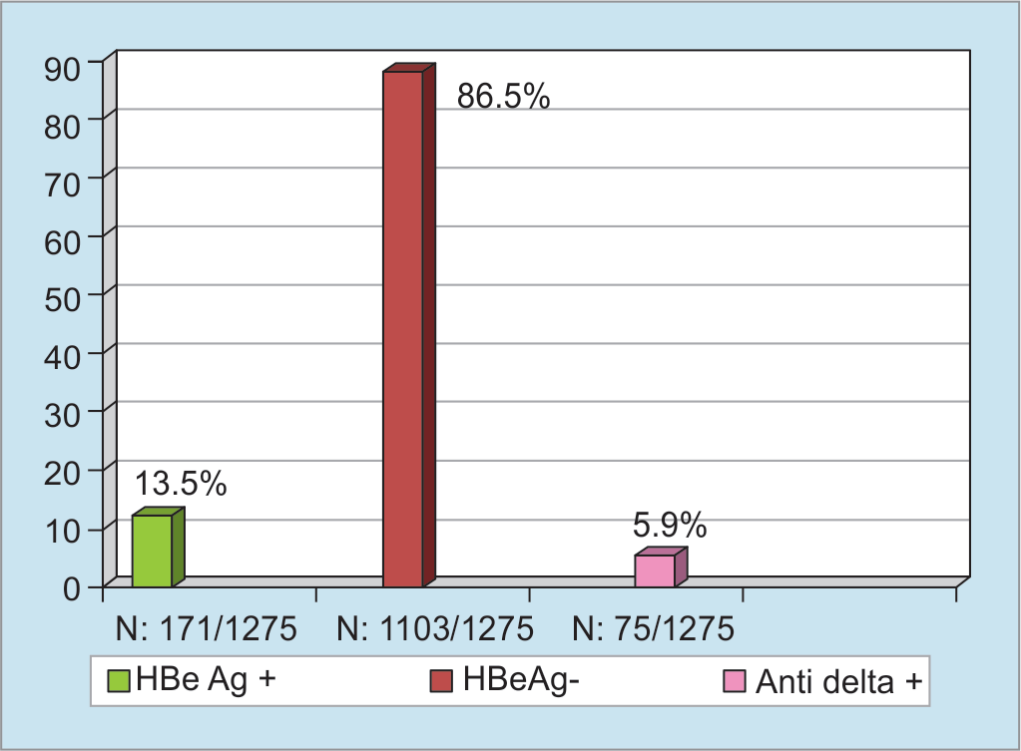
HBsAg (+) 1,275 cases (1998-2016)^5^

The estimated number of HBV carriers in Turkey is 3.3 million, with an overall HBV prevalence of 4.57%. Even a very conservative assessment means that 10% of the carriers would need treatment, which means that 330,000 chronic HBV cases would be eligible for treatment in Turkey alone. A total of 1,533 among 37,637 patients who were examined at the Ankara University School of Medicine Department of Gastroenterology were found to be HBsAg positive (4%) (Fig. 1, Graphs 2 and 3).

Treatment of CHB patients with active disease could reduce mortality related to liver disease among 330,000 chronic HBV cases by 80%.

It must be highlighted that viral hepatitis treatment is fully reimbursed in Turkey through the national insurance system, which covers all residents across Turkey.

In conclusion, several studies revealed an HBsAg positivity in less than 3.5% of the adult population and at least one-third of the population has been exposed to HBV infection in Turkey. The findings of population-based studies in Turkey with respect to the epidemiology of HBV and HCV revealed HBsAg positivity in 4%, anti-HCV positivity in 1%, and anti-HDV positivity in 2.8% of HBsAg-positive individuals.^4^
